# Chaotic Harris Hawks Optimization with Quasi-Reflection-Based Learning: An Application to Enhance CNN Design

**DOI:** 10.3390/s21196654

**Published:** 2021-10-07

**Authors:** Jameer Basha, Nebojsa Bacanin, Nikola Vukobrat, Miodrag Zivkovic, K. Venkatachalam, Stepan Hubálovský, Pavel Trojovský

**Affiliations:** 1 Department of Computer Science and Engineering, Hindusthan Institute of Technology, Coimbatore 641028, Tamil Nadu, India; jameer@hit.edu.in; 2 Faculty of Informatics and Computing, Singidunum University, Danijelova 32, 11000 Belgrade, Serbia; nikola.vukobrat.19@singimail.rs (N.V.); mzivkovic@singidunum.ac.rs (M.Z.); 3 Department of Applied Cybernetics, Faculty of Science, University of Hradec Králové, 50003 Hradec Králové, Czech Republic; venkatachalam.k@ieee.org (K.V.); stepan.hubalovsky@uhk.cz (S.H.); pavel.trojovsky@uhk.cz (P.T.)

**Keywords:** swarm intelligence, Harris Hawks optimization, exploitation–exploration trade-off, chaotic, quasi-reflection-based learning, convolutional neural networks, classification

## Abstract

The research presented in this manuscript proposes a novel Harris Hawks optimization algorithm with practical application for evolving convolutional neural network architecture to classify various grades of brain tumor using magnetic resonance imaging. The proposed improved Harris Hawks optimization method, which belongs to the group of swarm intelligence metaheuristics, further improves the exploration and exploitation abilities of the basic algorithm by incorporating a chaotic population initialization and local search, along with a replacement strategy based on the quasi-reflection-based learning procedure. The proposed method was first evaluated on 10 recent CEC2019 benchmarks and the achieved results are compared with the ones generated by the basic algorithm, as well as with results of other state-of-the-art approaches that were tested under the same experimental conditions. In subsequent empirical research, the proposed method was adapted and applied for a practical challenge of convolutional neural network design. The evolved network structures were validated against two datasets that contain images of a healthy brain and brain with tumors. The first dataset comprises well-known IXI and cancer imagining archive images, while the second dataset consists of axial T1-weighted brain tumor images, as proposed in one recently published study in the Q1 journal. After performing data augmentation, the first dataset encompasses 8.000 healthy and 8.000 brain tumor images with grades I, II, III, and IV and the second dataset includes 4.908 images with Glioma, Meningioma, and Pituitary, with 1.636 images belonging to each tumor class. The swarm intelligence-driven convolutional neural network approach was evaluated and compared to other, similar methods and achieved a superior performance. The obtained accuracy was over 95% in all conducted experiments. Based on the established results, it is reasonable to conclude that the proposed approach could be used to develop networks that can assist doctors in diagnostics and help in the early detection of brain tumors.

## 1. Introduction

As technology advances further every year, people are recognizing new means of solving certain problems with greater quality, precision, and efficiency. One of the technological domains that uncovered broad possibilities, and continues to do so, is artificial intelligence (AI). AI is not something that was revealed in the near past; it has existed for decades, but has only recently gained popularity among researchers and companies. The reason for this is the breakthrough in its processing power and storage capabilities, which increased the potential for more advanced AI applications. Today, almost every field uses some kind of AI, such as medicine, economy, marketing, etc. Moreover, most people are unaware that AI is influencing their life in multiple ways.

As AI is applied to a wide variety of fields, different methodologies and algorithms exist within this domain. Therefore, various AI taxonomies can be found in the modern literature; however, from the authors’ perspective, one of the most important taxonomies splits AI methods into two categories: metaheuristics and machine learning. Metaheuristics are problem-independent, high-level algorithms that provide a set of strategies to develop a heuristic for solving a particular problem and, as such, they cannot guarantee an optimum problem solution. However, they can obtain satisfying (in most cases, near-optimum) solutions in a rational amount of time [1].

Further, based on the type of phenomena that they emulate, metaheuristics can be divided into those that are inspired and those which are not inspired by nature [2]. Examples of non-nature inspired metaheuristics include tabu search (TS) [3] and differential evolution (DE) [4]. The two most prominent groups of nature-inspired metaheuristics include evolutionary algorithms (EA), which simulate the process of natural evolution, and swarm intelligence, which mimics a group of organisms from nature.

Swarm intelligence algorithms are known as efficient solvers of many NP-hard challenges [5]. Although many swarm algorithms have been devised in the recent decade, there is always a space for new ones, as well as for improvements in the existing ones, as a universal algorithm that can successfully tackle all problems cannot be created.

The main focus of the research proposed in this manuscript is the development of an enhanced Harris Hawks optimization (HHO) algorithm that addresses the observed flaws of its basic version. The HHO is a recently proposed, yet well-known swarm intelligence metaheuristics [6] that showed great potential in tackling many real-world challenges [7,8]. In this study, the authors tried to further investigate and expand the HHO’s potential by incorporating a chaotic mechanism and a novel replacement strategy that enhance both the exploitation and exploration of the basic algorithm, with only a small additional overhead in terms of computational complexity and new control parameters.

Established practice from the modern computer science literature states that when a new optimization method is devised, or an existing one is improved, it should first be validated on a more extensive set of so-called benchmarks and then applied to a practical problem. According to the no free lunch theorem (NFL), a method that performs equally well for all types of problems does not exist, and for some method to be tagged as well-performing, it should obtain a good average performance for most benchmark instances. Following this strategy, the proposed, enhanced HHO was first validated against a set of 10 novel CEC2019 bound-constrained benchmarks and, in these experiments, the near-optimum control parameter values, that obtain the best average results, are determined.

To further validate the proposed HHO, a method was adopted to tackle the practical issue of evolving convolutional neural network (CNN) hyper-parameters. One of the greatest challenges and obstacles in devising high-performing CNNs is the fact that a universal network, which will obtain a good performance for all problems, does not exist. Therefore, untrainable CNN parameters, which are known in the literature as hyper-parameters, should be finetuned to better fit certain optimization problems. Some of these parameters determine the overall CNN architecture (design); more precisely, they define the number and types of layer, along with their size in terms of the kernel, number of neurons and connections that are used. Other parameters determine the type of optimizer and activation function that is used, the learning rate value, etc. Noting this, there can be an infinite number of combinations in which one CNN can be structured, which itself represents one instance of NP-hard optimization problems.

The proposed HHO is utilized to find a proper CNN structure for the classification of magnetic resonance imaging (MRI) brain tumor grades. This specific dataset is chosen because the MRI brain tumor grades’ classification represents a very important task in the domain of medicine, which can potentially save lives and, therefore, the basic motivation behind this practical application is engaging efforts to further enhance the CNNs classification performance for this dataset and devise an "early warning" tool that will help doctors to diagnose brain tumors in early stages of development. Moreover, according to the literature survey, the potential of swarm intelligence for developing CNN structures for these types of data is not investigated enough. Rxamples of previous research from this domain are efforts shown in [9,10]. Thus, by evolving CNN structures for MRI tumor datasets, we aim to acccomplish the following objectives: first, to evaluate the proposed method for a specific practical problem and to try to improve the classification accuracy for this very important dataset.

In practice, it would be impossible to examine all possible hyper-parameters’ values, and for each hyper-parameter, lower and upper bounds are defined by the domain expert, as shown in [9]. In this way, by adjusting the search space boundaries, the evolved CNN’s structures target specific MRI brain tumor grade classification datasets, not any generic dataset.

It is noted that the practical application of the research shown in this manuscript represents the continuation of the investigation shown in [10]. Additionally, in the proposed study, other well-known swarm algorithms are also implemented for CNN design, and a broader comparative analysis is established. Most of these metaheuristics have not been evaluated for this challenge before.

Based on the above, the research proposed in this manuscript is guided by two basic investigation questions:

- Is it possible to further improve the basic HHO algorithm by addressing both search processes—exploitation and exploration?
- Is it possible to evolve a CNN that will establish better classification metrics for MRI brain tumor datasets than other state-of-the-art methods proposed in the modern literature?

The contribution of proposed study is three-fold:

- An enhanced version of the HHO metaheuristics has been developed that specifically targets the observed and known limitations and drawbacks of the basic HHO implementation;
- It was shown that the proposed method can generate high-performing CNN structures for MRI brain tumor classification that establish better performance metrics than other outstanding methods proposed in the literature;
- Other well-known swarm intelligence algorithms were tested for CNN design and a broader comparative analysis was performed.

The remainder of this paper is structured as follows: Section 2 provides a short overview of theoretical background relevant to proposed research along with literature review, Section 3 first provides basics of the HHO, then summarizes its drawbacks, and finally proposes the enhanced version of this promising algorithm. Section 4 and Section 5 are practical, where results of the proposed method along with comparative analysis with other state-of-the-art methods are first reported for standard CEC2019 benchmarks and then for CNN design for MRI brain tumor grade classification. The final Section 6 exhibits the closing remarks, and suggests possible future work in this field along with limitations of the performed study.

## 2. Preliminaries and Related Works

The goal of this section is to introduce readers to some basic concepts that serve as a background for this paper. Moreover, this section provides a literature review of the relevant topics.

### 2.1. Swarm Intelligence

Swarm intelligence algorithms come from a group of nature-inspired optimizers. These are population-based, iterative approaches that incrementally try to provide a solution for the problem at hand. Each individual from the population represents one potential solution, while communication between solutions is established by using some kind of indirect communication mechanism without a central component. The search process is guided by exploitation (intensification) and exploration (diversification) mechanisms, where the first one performs a search in the neighborhood of existing solutions and the latter explores previously undiscovered parts of the search space.

In most cases, it is possible to spot the natural phenomenon that algorithm simulates from its name, e.g., particle swarm optimization (PSO) [11], firefly algorithm (FA) [12], artificial bee colony (ABC) [13], seagull optimization algorithm (SOA) [14], bat algorithm (BA) [15], etc. [16,17].

With all advanced strategies of how the best swarm algorithms operate, they are successfully applied even outside research circles. Consequently, they make a huge contribution to real-world problems such as cloud computing and task scheduling [18,19], wireless sensor network localization and routing [20,21], numerous fields of medicine [22,23,24], prediction of COVID-19 cases [25], anomaly detection [26], etc. [27,28].

As the popularity of swarm intelligence metaheuristics grows, numerous different solutions are reported that enhance vast realms of AI methods and techniques. One of the most recent and prominent research fields is hybrid methods of swarm intelligence and various machine learning models adapted for a large number of real-world problems. Some practical machine learning challenges that are successfully tackled with swarm intelligence include feature selection [29,30,31], training artificial neural networks (ANNs) [32,33], text clustering [34] and many others [35,36].

### 2.2. Convolutional Neural Networks

The CNNs are well-known and widely utilized methods from the deep learning area, and they are capable of providing outstanding results in different application domains. The CNNs have achieved outstanding results in the areas of computer vision, speech recognition, and natural language processing (NLP) [37,38,39,40].

The CNN model is based on the visual cortex of the brain, which consists of several layers. This structure starts with the input layer, and every subsequent layer receives the outputs of the previous layer as its inputs, processes the information, and sends it to the next layer. After each of the layers in the network, data become more filtered. In this way, each layer produces output more detailed data, while the first layer can process more easily, without losing important features. Layers in the CNN structure are separated into three groups: convolutional, pooling, and fully connected (or dense).

Convolutional layers are responsible for filtering the data by utilizing the convolutional operation and extracting the features in a smaller size than the input. Typical sizes include 3 × 3, 5 × 5, and 7 × 7. Convolutional function over the input vector can be mathematically represented by Equation (1):

(1) $$z_{i,j,k}^{\left[l\right]}=w_{k}^{\left[l\right]}x_{i,j}^{\left[l\right]}+b_{k}^{\left[l\right]},$$

where $z_{i,j,k}^{\left[l\right]}$ represents value of the output feature of the *k*-th feature map at position i,j and layer *l*. Representation of the input at location i,j is given as *x*; *w* represents the filters, while the bias is denoted as *b*.

After convolution, the activation is performed, by using the following expression:

(2) $$g_{i,j,k}^{\left[l\right]}=g\left(z_{i,j,k}^{\left[l\right]}\right)$$

where the g(·) denotes the non-linear function using the output.

Pooling layers have the task of reducing the resolution. Two commonly used variants of pooling layer implementations are max and average pooling. The function that defines the pooling is given with Equation (3).

(3) $$y_{i,j,k}^{\left[l\right]}=pooling\left(g_{i,j,k}^{\left[l\right]}\right).$$

where *y* denotes the output of the pooling operation.

Finally, fully connected layers have the task of carrying out the classification process. Classification is performed by the softmax layer in the case of multi-labeled datasets, or by the logistic layer (sigmoidal) in the case of binary classification.

The CNN training is usually executed by utilizing gradient-descent-based methods [41]. The goal of these optimizers is to minimize the loss function over a series of steps called epochs. In each epoch, network weights and biases are adjusted, so the loss function is minimized. Again, in the literature, as well as in practical applications, many loss functions exist and some of the most commonly employed are binary and categorical cross-entropy *H* for binary and multi-label classification tasks, respectively. The function is specified by two distributions *p* and *q* across discrete variable *x*, as shown in Equation (4).

(4) $$H\left(p,q\right)=-\sum_{x}p\left(x\right)ln\left(q\left(x\right)\right)$$

One of the problems that CNNs face is creating a model that will perform well on both the training set and the new data. In many practical uses, the model performs well on the training data; however, it fails to establish a satisfying classification accuracy on the testing data. This problem is known in the literature as over-fitting. Many methods have been proposed to address this issue; however, one of the most commonly used is the dropout technique [42], which falls in the domain of regularization. Dropout is not considered computationally expensive, but is a very efficient approach that prevents the over-fitting problem by the random removal of some neurons in the fully connected layer during the training process.

The standard, traditional way to determine the appropriate CNNs design, as well as a classic ANN architecture, for a given problem is to evaluate them by first performing training on the train set and then executing testing on previously unseen data. As already noted in Section 1, this represents a major issue in this domain, which is known as the tuning of hyper-parameters (optimization). Unfortunately, this procedure is lengthy and time-consuming; moreover, it is based on "trial and error" and requires human intervention. Recently, many automated frameworks based on metaheuristics that generate CNN structures for a given task, are devised [43]. Due to the NP-hard nature of the hyper-parameters’ optimization challenge, metaheuristics, especially swarm intelligence, proved very efficient methods for devising such frameworks [44,45,46].

It should also be emphasized that the HHO algorithm has already been implemented to optimize ANN and CNN hyperparameters in previous research [7,47]. However, since every problem is specific, and by taking the NFL into account, the HHOs potential to evolve a CNN’s structure to classify MRI brain tumor images has not been established.

### 2.3. MRI Brain Tumor Grades Classification

Glioma tumors are the most frequent type of brain tumor in the adult population [48]. They are classified into four grades, corresponding to severity levels ranging from I to IV [49]. Gliomas classified as grade I are considered benign, while grade IV gliomas represent malignant tumors that spread fast. One of the key factors of the successful treatment of patients with glioma is a fast diagnostics and early detection [50]. However, in practice, this procedure is complicated and includes magnetic resonance imaging (MRI), followed by invasive biopsy if a tumor is suspected. It can take weeks, sometimes months, for a procedure to finish and to obtain a decisive answer. To speed up the process, the usage of computer-aided tools is necessary to help doctors interpret the MRI images and classify the grade of the tumors [51,52].

For tumor diagnosis, MRI is the only non-invasive methodology that provides valuable data in the shape of 2D and 3D images. The CNNs are known and proven classifiers that can be utilized to help in the identification of objects in MRI images and perform segmentation. However, since the MRI dataset is specific and different from other, especially generic, datasets, the CNN with a specific design should be evolved, and that is why some most recent research from this domain utilizes MRI paired with CNNs and swarm intelligence metaheuristics [10,53].

## 3. Proposed Method

In the beginning, this section introduces the basic HHO. Then, it points out the noticed deficiencies of original HHO implementation and, finally, the modifications that target the observed flaws of the basic HHO are proposed at the end of this section.

### 3.1. Original HHO

The HHO algorithm, as its name states, is inspired by different Harris Hawks’ strategies during their attacks on prey in nature. These attacking phases consist of three steps: exploration, the transition from exploration to exploitation, and, finally, the exploitation phase. The algorithm was initially proposed by Heidari et al. in 2019 [6].

In the exploration phase, the HHO algorithm strives to find the closest solution to the global optimum. During this phase, an algorithm is randomly initialized on multiple locations and, step-by-step, moves closer to its prey, mimicking how hawks attack in their natural surroundings. To achieve this efficiently, the HHO utilizes two strategies with equal probabilities, determined with parameter *q* as follows [6]:

(5) $$X\left(t+1\right)=\left\{\begin{array}{c} X_{rand}\left(t\right)-r_{1}\left|X_{rand}\left(t\right)-2r_{2}X\left(t\right)\right|,q\geq 0\\ \left(X_{best}\left(t\right)-X_{m}\left(t\right)\right)-r_{3}\left(LB+r_{4}\left(UB-LB\right)\right),q<0.5, \end{array}\right.$$

where *q*, together with r1, r2, r3 and r4, represent random numbers from the range [0,1], which are updated in each iteration, X(t+1) is the solutions’ position vector for next iteration, while $X_{best}\left(t\right)$, X(t) and $X_{m}\left(t\right)$ denote the best, current and average solutions’ positions in the ongoing iteration *t*. Finally, LB and UB are lower and upper bounds of variables that define the scope of solutions in the search space. Furthermore, to obtain an average position of the solutions $X_{m}\left(t\right)$, the simplest averaging approach is employed:

(6) $$X_{m}\left(t\right)=\frac{1}{N}\sum_{i=1}^{N}X_{i}\left(t\right),$$

where *N* presents the total number of solutions and $X_{i}\left(t\right)$ is location (position) of individual *X* at iteration *t*.

During the exploration phase, the HHO can change from exploitation to exploration for different amounts of time, depending on the strength of the solution (prey energy). The strength of solution updates in each iteration is as follows:

(7) $$E=2E_{0}\left(1-\frac{t}{T}\right),$$

where *T* expresses the maximal number of rounds (iterations) in a run and $E_{0}$ initial strength of pray energy, which changes randomly inside the [−1,1] interval.

During the exploitation phase, the hawk attacks its prey. However, the prey tries to escape, so the hawk needs to change between different strategies in order to exhaust and consequently catch the prey. In a real situation, hawks will try to come closer and closer to the prey, to make it easier for them to catch it. To incorporate this into the optimization algorithm, they change their attacking pattern from softer to harder. When |E|≥0.5, a soft besiege is applied, while when |E|<0.5, hard besiege occurs.

When r≥0.5 and |E|≥0.5, the prey still has energy, so hawks will encircle softly to make the prey more exhausted. This behavior is modeled with the following expressions  [6]:

(8) $$X\left(t+1\right)=\Delta X\left(t\right)-E|JX_{best}\left(t\right)-X\left(t\right)|$$

(9) $$\Delta X\left(t\right)=X_{best}\left(t\right)-X\left(t\right),$$

where ΔX(t) is a vector difference between the best solution (prey) and solution position in iteration *t*. Attribute *J* is randomly changed in each round and simulates the target escaping strategy:

(10) $$J=2(1-r_{5}),$$

where $r_{5}$ is randomly generated number in interval [0,1]). In a case when r≥0.5 and |E|<0.5, prey is exhausted and hawks can perform a hard attack. In this case, the current positions are updated as:

(11) $$X\left(t+1\right)=X_{best}\left(t\right)-E\left|\Delta X\left(t\right)\right|$$

Furthermore, in the situation when the prey still has some energy available, a soft push is performed before the hawks’ attack can occur. This form of attack utilizes zig-zag movements known as leapfrog movements, which are common in nature. To perform this, the hawk can evaluate the following rule:

(12) $$Y=X_{best}\left(t\right)-E\left|JX_{best}\left(t\right)-X\left(t\right)\right|,$$

and then dive in a leapfrog pattern as follows:

(13) Z=Y+S×LF(D),

where *D* is the dimension of a problem and *S* represents a random vector of 1×D size, while LF is levy flight function, calculated as:

(14) $$LF\left(x\right)=0.01\times \frac{u\times \sigma }{{\left|v\right|}^{\frac{1}{\beta }}},\sigma ={\left(\frac{\Gamma \left(1+\beta \right)\times sin\left(\frac{\pi \beta }{2}\right)}{\Gamma \left(\frac{1+\beta }{2}\right)\times \beta \times 2^{\left(\frac{\beta -1}{2}\right)}}\right)}^{\frac{1}{\beta }}$$

Hence, the strategy for updating individuals’ positions is calculated as follows:

(15) $$X\left(t+1\right)=\left\{\begin{array}{c} Y,\;\mathrm{if}\;F(Y)<F(X(t))\\ Z,\;\mathrm{if}\;F(Z)<F(X(t)), \end{array}\right.$$

where *Y* and *Z* are calculated by utilizing Equations (12) and (13).

Finally, in the r≤0.5 and |E|<0.5 case (hard besiege with progressive rapid dives), the prey does not have energy, and a hard attack is utilized before hawks hunt down the prey. Therefore, hawks decrease their average distance from the pray using the following strategy:

(16) $$X\left(t+1\right)=\left\{\begin{array}{c} Y,\;\mathrm{if}\;F(Y)<F(X(t))\\ Z,\;\mathrm{if}\;F(Z)<F(X(t)), \end{array}\right.$$

where, contrary to Equation (15), *Y* and *Z* are obtained using the following rules:

(17) $$Y=X_{best}\left(t\right)-E\left|JX_{best}\left(t\right)-X\left(t\right)\right|$$

(18) Z=Y+S×LF(D)

In the modern literature from the domain of swarm intelligence, the two most commonly used methods for calculating algorithms’ complexity are established: the first one only accounts for the number of fitness function evaluations (FFEs) because this operation is the most expensive in terms of computational resources  [54] and the second one, besides FFEs, also includes the cost of updating solutions’ positions [6,17,55]. The cost of generating the initial population is excluded because it is relatively inexpensive compared to the cost of the solutions’ update process. Both methods calculate complexity in terms of *T*; however, if the total number of FFEs is taken as the termination condition when comparing the performance of different algorithms, a comparison in terms of complexity is not needed.

Following the second method, as was suggested in [6], the complexity of the original HHO is described as follows:

- Number of FFEs in the initialization phase-O(N)
- Number of FFEs in the solutions’ updating phase-O(N·T)
- Cost of solutions’ updating phase-O(N·D·T)

Taking all the above into account, the computational complexity of the HHO is derived as follows:

(19) O(HHO)=O(N)+O(N·T)+O(N·D·T)=O(N·(T+T·D+1))

More details about HHO can be captured from [6].

### 3.2. Motivation for Improvements and Proposed Enhanced HHO Method

Notwithstanding the outstanding performance of basic HHO [6], by conducting simulations with standard CEC bound-constrained benchmarks, it was noted that the original version can be further improved by addressing both processes—exploitation and exploration.

For some testing instances, especially with a higher number of dimensions, it happens that, in early iterations, the algorithm gets stuck in sub-optimal domains of the search space. After analyzing the diversity of convergence and solutions in such scenarios, it was concluded that if the “early” best solutions miss the right part of the search space, then most of the solutions also converge there and it is hard for the HHO to “escape” from this region. In other words, solution diversity for some problems is not satisfying in early execution cycles, and that is extremely dangerous if most of the solutions are generated far from the optimum domain. However, after some iterations, by performing HHO exploration, promising domains are discovered. However, this usually happens in the final stages of execution, when it is late for the search process to perform fine-tuning in this region, and consequently, less good solutions are generated at the end of a run.

The above-mentioned limitation of the basic HHO can also be viewed from the perspective of exploration–exploitation balance. Namely, in early iterations, this trade-off is biased towards exploitation, while in later iterations, when it should move towards exploitation, the intensification–diversification trade-off is in an equilibrium.

To address the above-mentioned issues in the original HHO, this study proposes three modifications.

The first modification is chaos-based population initialization. The idea of embedding chaotic maps in metaheuristics algorithms was first introduced by Caponetto et al. in [56]. The stochastic nature of most metaheuristics approaches is based on random number generators; however, many recent studies have shown that the search process can be more efficient if it is based on chaotic sequences [57,58].

Chaos is defined as non-linear movements of the dynamic systems that exhibit ergodicity and stochasticity, and are susceptible to initial conditions. Generation of the population by chaotic sequences has previously been used in multiple metaheuristics approaches in various domains. Some examples include chaotic multi-swarm whale optimizer for boosting the support vector machine (SVM) that assists doctors in medical diagnostics [57], chaos-enhanced firefly metaheuristics, applied to the mechanical design optimization problem [59], K-means clustering algorithm enhanced with chaotic ABC [60], and many others [58,61,62].

Many chaotic maps are available, such as the circle map, Chebyshev map, intermittency map, iterative map, logistic map, sine map, sinusoidal map, tent map, and singer map. After performing experiments with all the above-mentioned chaotic maps, the best results were achieved with a logistic map, and this was chosen for implementation in HHO.

The chaotic search is implemented in the proposed HHO by generating the chaotic sequence in accordance with the constraints of the observed problem, and then the generated sequence is used by individuals for exploration of the search space. The proposed method utilizes the chaotic sequence β, that starts from the initial random number $\beta_{0}$, generated by the logistic mapping, according to the Equation (20):

(20) $$\beta_{i+1}=\mu \beta_{i}\times \left(1-\beta_{i}\right),i=1,2,\cdots ,N-1,$$

where μ and *N* are the chaotic control parameter and size of population, respectively. The μ usually has the value 4 [62], as was also set in this study, to ensure chaotic movements of individuals, while $0<\beta_{0}<1$ and $\beta_{0}\neq 0.25,0.5,0.75,1$.

The process of mapping solutions to generated chaotic sequences is accomplished with the following expression for each parameter *j* of individual *i*:

(21) $$X_{i}^{c}=\beta_{i}X_{i},$$

where $X_{i}^{c}$ is new position of individual *i* after chaotic perturbations.

Taking all these into account, the details of chaotic-based population initialization are provided in Algorithm 1.
**Algorithm 1** Pseudo-code for chaotic-based population initializationStep 1: Generate standard random population *P* of *N* solutions with expression: $X_{i}=LB+\left(UB-LB\right)\cdot rand\left(0,1\right),\;\;i=1,...N$, where rand(0,1) is pseudo-random number drawn from the interval [0,1].Step 2: Generate chaotic population $P^{c}$ of *N* individuals by mapping solutions from *P* to chaotic sequences using expressions (20) and (21).Step 3: Calculate fitness of all solutions from *P* and $P^{c}$.Step 4: Sort all solutions from $P\cup P^{c}$ according to fitness.Step 5: Select *N* best individuals from sorted set $P\cup P^{c}$ as initial population.

In this way, the quality of solutions is improved at the beginning of a run and the search agents may utilize more iterations for exploitation. However, despite the efficient chaotic-based initialization, when tackling challenges with many local optima, metaheuristics may still suffer from premature convergence. As noted above, the exploration of basic HHO, which is crucial for avoiding premature convergence, should be improved, which motivated the introduction of the second modification.

One of the most efficient available strategies for improving both intensification and exploration, as well as its balance, is the quasi-reflection-based learning (QRL) procedure [63]. By using the QRL, quasi-reflexive-opposite solutions are generated and if, for example, the original individual’s position is far away from the optimum, there is a good chance that its opposite solution may be in the domain in which an optimum resides.

By applying the QRL procedure, the quasi-reflexive-opposite individual $X^{qr}$ of the solution *X* is generated in the following way:

(22) $$X^{qr}=\mathrm{rnd}\left(\frac{LB+UB}{2},X\right),$$

where $\mathrm{rnd}\left(\frac{LB+UB}{2},X\right)$ generates random number from uniform distribution in range $\left[\frac{LB+UB}{2},X\right]$. This procedure is executed for each parameter of solution *X* in *D* dimensions.

The proposed improved HHO adopts a simple replacement strategy of the worst and best individuals from the population based on the QRL. Applying the QRL mechanism yields the best performance in early iterations by significantly improving exploration if the current worst solution ($X_{worst}$) is replaced with the quasi-reflexive-opposite best individual ($X_{best}^{qr}$). However, based on empirical research, in later iterations, when exploitation should be intensified, it is better if the $X_{best}$ is replaced with its quasi-reflexive-opposite. In both cases, a greedy selection is applied and a solution with a higher fitness value is kept in the population for the next iteration.

Finally, the third modification that was incorporated into the basic HHO is chaotic search (CS) strategy [64] around the $X_{best}$ solution. During practical experiments, it was noted that, for very challenging benchmarks, e.g., multi-modal shifted and rotated functions [65], in late iterations, the $X_{best}$ may become “trapped” in one of the local optima and, in such scenarios, the QRL mechanism is not able to generate $X_{best}^{qr}$ with better fitness than the original $X_{best}$. The consequence of such scenarios is a quality of worse solutions at the end of a run and, consequently, worse mean values.

The abovementioned case is mitigated by employing the CS strategy in the following way: in later iterations, if the $X_{best}$ cannot be improved in best threshold (bt) fitness function evaluations (FFEs), instead of generating $X_{best}^{qr}$, the CS is performed around the $X_{best}$. The CS strategy for generating the chaotic current best ($X_{best}^{c}$) is described with the following equations:

(23) $$X_{best}^{c}=\left(1-\theta \right)X_{best}+\theta S$$

(24) $$S=LB+\beta_{best}\left(UB-LB\right)$$

where $\beta_{best}$ is a new chaotic sequence for the $X_{best}$ determined by Equation (20) and θ is dynamic shrinkage parameter that depends on the current FFEs and the maximum number of fitness function evaluations (maxFFEs) in the run:

(25) $$\theta =\frac{maxFFEs-FFEs+1}{maxFFEs}$$

Dynamic λ enables a better exploitation–exploration trade-off by establishing wider and narrower search radius around the $X_{best}$ in lower and higher iterations, respectively. The FFEs and maxFFEs can be replaced with *t* and *T* when the total number of iterations in a run is considered the termination condition.

Finally, to control which of the proposed two QRL or the CS strategies will be triggered, another control parameter, adaptive behavior, denoted as ψ, is introduced. This procedure is mathematically described with the following three expressions:

(26) $$X_{worst}=X_{best}^{qr},\;\;\;\;\mathrm{if}\;\;\;\;\psi <FFEs$$

(27) $$X_{best}=X_{best}^{qr},\;\;\;\;\mathrm{if}\;\;\;\;\psi \geq FFEs\;\;\;\mathrm{and}\;\;\;k<bt$$

(28) $$X_{best}=X_{best}^{c},\;\;\;\;\mathrm{if}\;\;\;\;\psi \geq FFEs\;\;\;\mathrm{and}\;\;\;k\geq bt,$$

where *k* is incremented every time $X_{best}$ cannot be improved by QRL and bt stands for the predefined $X_{best}$ improvement threshold. The FFEs simply represents the current number of fitness function evaluations and can be replaced with *t* if the number of iterations is taken as the termination condition. The above expression will execute if, and only if, the newly generated solution is better than the current solution, according to the greedy selection mechanism.

Since metaheuristics are stochastic methods in which randomness plays an important part, empirical simulations are the most reliable way of determining how the control parameters’ values affect the search process. Additionally, in the case of the proposed enhanced HHO, as shown in Section 4, the values of control parameters ψ and bt, that, on average, obtain the best performance for a wider set of benchmarks, are empirically determined.

Notwithstanding that the enhanced HHO metaheuristics adopts chaotic population initialization, QRL replacement, and CS strategies, for the sake of simple naming conventions, the proposed method is titled enhanced HHO (eHHO) and its working details, in a form of pseudo-code, are provided in Algorithm 2.
**Algorithm 2** Proposed eHHO pseudo-codeInputs: The population size *N* and maximum FFEs number (maxFFE)Initialize population $X_{i}$, (i=1,2,3,…N) according to Algorithm 1Initialize FFEs=0**while**FFEs≤maxFFEs**do** Calculate the fitness values of all individuals Set $X_{best}$ as the location of current best solution **for** each solution $X_{i}$ **do**  Update the initial energy $E_{0}$ and jump strength *J*  Update *E*  **if** |E|≥1 **then**   Exploration phase   Update the location vector  **end if**  **if** |E|<1 **then**   Exploitation phase   **if** r≥0.5 and |E|≥0.5 **then**    Soft besiege    Update the location vector by soft besiege   **else if** r≥0.5 and |E|<0.5 **then**    Hard besiege    Update the location vector by using hard besiege   **else if** r<0.5 and |E|≥0.5 **then**    Soft besiege with progressive rapid dives    Update the location vector by using soft besiege with rapid dives   **else if** r<0.5 and |E|<0.5 **then**    Hard besiege with progressive rapid dives    Update the location vector using hard besiege with progressive rapid dives   **end if**  **end if** **end for** **if** ψ<FFEs **then**  Generate $X_{best}^{qr}$  Perform greedy selection between $X_{worst}$ and $X_{best}^{qr}$ **else if** ψ≥t and k<bt **then**  Generate $X_{best}^{qr}$  Perform greedy selection between $X_{best}$ and $X_{best}^{qr}$ **else**  Generate $X_{best}^{c}$  Perform greedy selection between $X_{best}$ and $X_{best}^{c}$ **end if****end while**Return $X_{best}$Post-process results and visualization

As shown in Section 4 and Section 5, the proposed eHHO managed to achieve a better performance than the basic HHO. However, according to the NFL theorem, there is always a trade-off. The proposed eHHO employs additional control parameters ψ and bt and performs *N* more, and one more FFEs, during initialization and throughout its iterations, respectively. Moreover, the eHHO updates one more solution in each iteration.

Following the same approach as that used to calculate computational complexity, as well as the costs (Equation (19)), in terms of *T*, the eHHO complexity can be expressed as:

(29) O(eHHO)=O(2·N)+O((N+1)·T)+O((N+1)·D·T)

## 4. CEC2019 Benchmark Functions Experiments

According to good experimental practice, the proposed approach was verified with the standard unconstrained benchmark functions, set before application to a concrete practical task. The introduced eHHO method was evaluated on the 10 recent benchmark function set, introduced in the Congress on Evolutionary Computation 2019 (CEC2019) [66], and the obtained experimental results were compared to other modern approaches, which are followed by the standard statistical tests.

The CEC2019 bound-constrained benchmark function details are provided in Table 1.

### 4.1. Experimental Setup and Control Parameters’ Adjustments

The performance of the suggested eHHO metaheuristics was evaluated by comparative analysis with the basic HHO, and nine other modern metaheuristics approaches, namely elephant herding optimization (EHO) [67], EHO improved (EHOI) [68], salp swarm algorithm (SSA) [69], sine cosine algorithm (SCA) [70], grasshopper optimization algorithm (GOA) [55], whale optimization algorithm (WOA) [17], biogeography-based optimization (BBO) [71], moth–flame optimization (MFO) [72] and particle swarm optimization (PSO) [11].

The simulation results obtained with the above-mentioned algorithms for the same benchmarks were reported in [68]. However, in the study proposed in this manuscript, experiments are recreated to validate the results of [68] and establish a more objective comparative analysis. The methods in [68] were tested with *N* = 50 and *T* = 500, and this setup may lead to a biased comparative analysis because not all algorithms utilize the same number of FFEs in one iteration. Therefore, in this study, the termination condition for all approaches was set according to the total utilized FFEs, and to establish similar conditions, as in [68], the maxFFEs was set to 25,000 (50×500).

The results are reported for 50 separate runs, while the average (mean) and standard deviation (std) results were taken as performance metrics. The value of dynamic behavior parameter ψ was determined empirically and set to maxFFE/4; in this case, to 6250 (25,000/4). Similarly, by performing extensive simulations, the mathematical expression for calculating bt value is derived: round((maxFFE−ψ)/(10·N)), where round represents a standard function that rounds input to the closest integer. In this case, the bt was set to 38 ((25,000–6250)/10×50). Other parameters are adjusted as is suggested in [6]. All other HHO and eHHO control parameters are dynamic, and their values are adjusted throughout the run according to the FFEs and maxFFEs. For more details, please refer to Section 3.1.

Control parameters for other metaheuristics included in the analysis were adjusted as suggested in the original manuscript.

A brief summary of eHHO control parameters used throughout the conducted experiments is given in Table 2.

### 4.2. Results and Discussion

The results from the ten conducted CEC2019 simulations are presented in Table 3. The reported results are shown as scientific notations, and the best mean values for each of the benchmark instances are shown in bold for easier reading. It is noted similar performance metrics to those reported in [68] were obtained.

A comparative analysis of the results provided in Table 3 indicates that the upper-level performance of the suggested eHHO approach is better than the other compared algorithms. For the majority of CEC2019 functions (more precisely, eight out of ten), eHHO achieved the best mean values. The exceptions include CEC03 benchmark, where all algorithms obtained the same mean value, and CEC07 test instance, where the BBO, on average, managed to achieve the best quality of solutions. Additionally, it can be noted that the eHHO algorithm drastically improves the performance of basic HHO metaheuristics, which obtained mediocre results compared to other approaches, justifying the goal of devising an enhanced HHO algorithm.

In modern computer science theory, when comparing different algorithms, it is typically not enough to declare that one algorithm is better than others only in terms of the obtained results. It is also necessary to determine if the generated improvements are significant in terms of statistics. Therefore, a Friedman test [73,74] and two-way variance analysis by ranks were employed to determine if there is a significant difference in the results of the proposed eHHO and other methods encompassed by the comparison. The Friedman test results for the 11 compared algorithms over the 10 CEC19 functions are presented in Table 4.

The results presented in Table 4 statistically indicate that the proposed eHHO algorithm has a superior performance when compared to the other ten algorithms, with an average rank value of 1.6. The basic HHO obtained an average ranking of 6.4. Additionally, the Friedman statistics ($\chi_{r}^{2}=51.4$) is larger than the $\chi^{2}$ critical value, with 10 degrees of freedom (18.3), at the significance level α=0.05. As the result, the null hypothesis ($H_{0}$) can be rejected, and it can be stated that the suggested eHHO achieved significantly different results to the other ten algorithms.

Furthermore, the research published in [75] reports that the Iman and Davenport’s test [76] could provide more precise results than the $\chi^{2}$. Therefore, Iman and Davenport’s test was also conducted. After calculations, the result of the Iman and Davenport’s test is 9.53E+00, which is significantly larger than the critical value of the *F*-distribution (F(9,9×10)=1.93E+00). Consequently, Iman and Davenport’s test also rejects $H_{0}$. The p−value is smaller than the significance level for both the executed statistical tests.

Results for Friedman and Iman and Davenport’s test with α=0.05 are summarized as follows: Friedmanvalue=5.14E+01, $\chi^{2}=1.83E+01$, Iman−Davenportvalue=9.53E+00, Fcriticalvalue=1.93E+00 and p−values of 1.11E−16 and 1.11E−13.

Since the null hypothesis was rejected by both performed statistical tests, the non-parametric post-hoc procedure, Holm’s step-down procedure, was also conducted and presented in Table 5. By using this procedure, all methods are sorted according to their *p* value and compared with α/(k−i), where *k* and *i* represent the degree of freedom (in this work k=10) and the algorithm number after sorting, according to the *p* value in ascending order (which corresponds to rank), respectively. In this study, the α is set to 0.05 and 0.1. It is also noted that the *p*-value results are provided in scientific notation.

The results given in the Table 5 suggest that the proposed algorithm significantly outperformed all opponent algorithms, except EHOI, at both significance levels α=0.1 and α=0.05.

**Figure 1:**
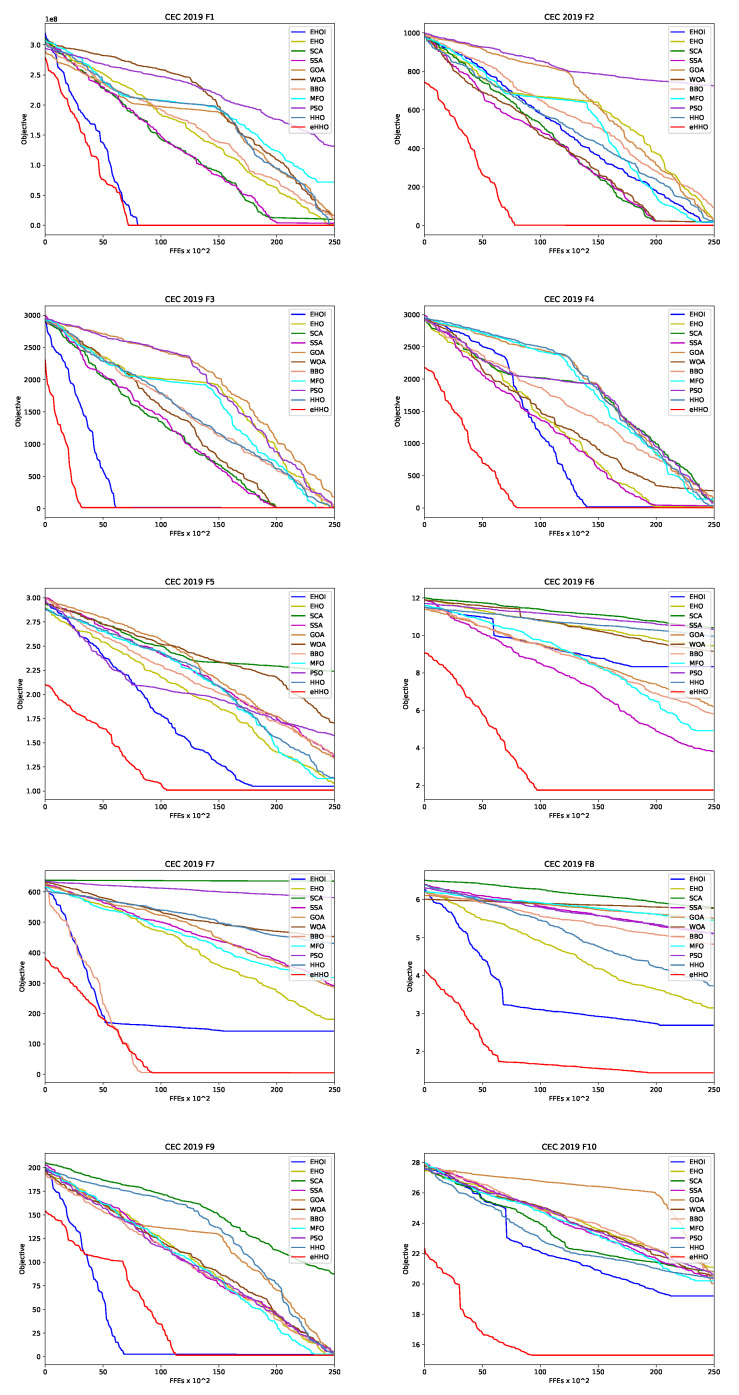
Graphs for convergence speed comparison for 10 CEC2019 benchmark functions—eHHO vs. other approaches.

An average convergence speed comparison between proposed eHHO and 10 other metaheuristics included in the analysis is given in Figure 1. From the presented graphs, the superiority of the devised approach compared to other methods can be categorically validated, and a few interesting things can be noticed. First, the effect of chaotic-based population initialization on the search space exploration is evident—this mechanism enables eHHO to better identify the boundaries of the optimum search domain at the beginning of a run. Second, in most challenges, during ψ FFEs, the eHHO managed to converge to near optimum levels and after this, a fine-tuned exploitation, that further improved the results’ quality, was performed. However, for problem CEC09 6250 FFEs was not enough to find a proper part of the search space, which can be clearly seen in the graph. In this case, somewhere between 5000 aQ!nd 6500 FFEs, the search process was “stuck”; however, when the CS is triggered, it converges relatively smoothly towards an optimum.

Finally, to demonstrate the effects of the control parameter ψ on the search process, additional experiments with varying ψ values are conducted by taking ψ increments of 6250 FFEs. Therefore, besides ψ=6250, as the most promising value for CEC2019 benchmarks, ψ was adjusted to 0, 12,500, 18,750, and 25,000 in four additional simulations. In the first case, when ψ=0, the greedy selection between $X_{worst}$ and $X_{best}^{qr}$ is be performed in each iteration, and the exploration process is amplified during the whole run. However, in the last case, when ψ = 25,000, the greedy selection between $X_{best}$ and $X_{best}^{qr}$ or $X_{best}$ and $X_{best}^{c}$ is executed, favoring intensification around the $X_{best}$. Mean values, calculated over 50 independent runs with varying ψ for CEC2019 simulations, are summarized in Table 6, where the best results are marked with bold font.

From the presented table with varying ψ values, a few interesting things can be observed. First, according to the NFL theorem, there are no universal control parameters’ values that can obtain the best results for all benchmark instances, and that is why the results generated with control parameter values that establish the best average quality of solutions are reported. In this case, for seven out of the ten benchmarks, the proposed eHHO obtains the best performance when ψ=6.250, while, for two benchmark instances, it was determined that the best performance is achieved when ψ=12.500. For the test instance, with CEC03 for all ψ settings, the same result is achieved. These statistics are provided in the last row of Table 6.

Further, it can be observed that the worst results are generated with ψ=25.000 (the only exception isthe CEC05 test instance). When ψ is adjusted to 25.000, the eHHO in each iteration performs exploitation around the current best solution by using either QRL or CS. Since the basic HHO suffers from poor exploration in early cycles, by conducting exploitation around the $X_{best}$ throughout the whole run, the mean results are worse. This is because the algorithm often misses promising regions of the search space in early iterations.

It also can be noted that the eHHO outscores BBO in CEC10 benchmark when ψ=12.500, while, with ψ set to 6.250, the BBO outperforms the method proposed in this study, as shown in Table 3. However, the only objective comparative analysis can be performed when the same control parameter values are used for all benchmark instances, and the above-mentioned remark cannot be taken into account when comparing the quality of the results the proposed eHHO with other state-of-the-art approaches.

**Figure 2:**
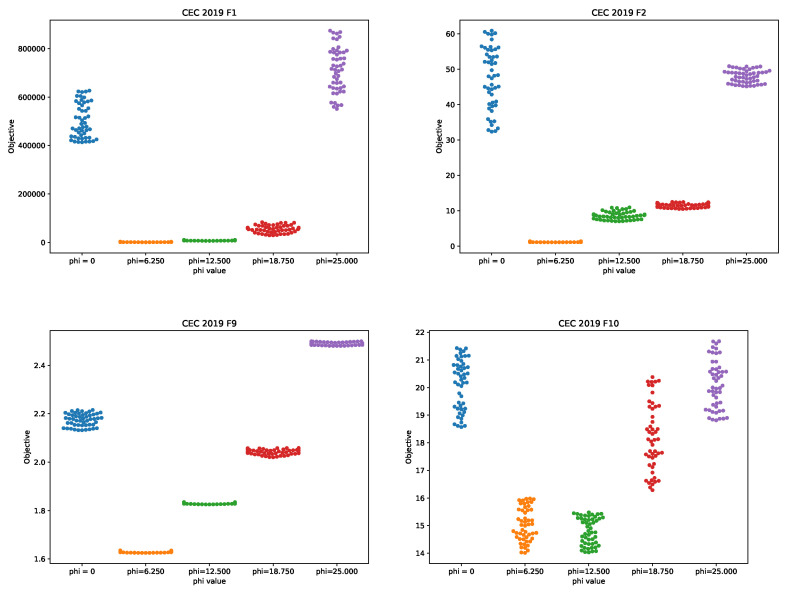
The eHHO swarm plots for CEC01, CEC02, CEC09 and CEC10 benchmarks with varying ψ value.

Finally, to better visualize eHHO performance with varying ψ values, swarm plot diagrams for some functions over 50 runs are shown in Figure 2.

## 5. CNN Design for MRI Brain Tumor Grades Simulations

After validating the proposed eHHO on the CEC2019 bound-constrained benchmarks, following good practice from the literature, simulations for the practical CNN design challenge were performed. The generated CNN structures were evaluated for classification tasks on two MRI brain tumor grades datasets.

The CNN hyperparameters’ optimization study adopts a similar approach as in [9]. Instead of training and evaluating millions of possible CNNs architectures, e.g., by using a grid search, the appropriate architectures were evolved by evaluating only a few hundred potential candidates using the guided eHHO approach, which leads to a drastic decrease in the overall computational costs. As in the referenced paper [9], this research utilizes the dropout technique [42] as the regularization option. This method is not considered computationally expensive, yet it is a very efficient approach that prevents the over-fitting problem by the random removal of some neurons in the fully connected layer during the training process.

All implementations are conducted in Python with TensorFlow and Keras libraries, along with scikitlearn, numpy and pandas, while matplotlib and seaborn libraries are used for visualization. Simulations are conducted on Intel®i7 platform with six × NVidia Geforce RTX 3080 GPUs.

This section first provides information about datasets and pre-processing, followed by the basic simulation setup (employed hyper-parameters, algorithm adaptations, and flow-chart) and the obtained results, along with a comparative analysis.

### 5.1. Datasets Details and Pre-Processing

Similarly, as in [9,10], two datasets are used in experiments. The first dataset (dataset1) is comprised of 600 normal brain images retrieved from the IXI database [77] and of 591 glioma brain tumor images with four grades (130 retrieved from REMBRANDT repository [78], 262 captured from TCGA-GBM dataset [79] and 199 downloaded from TCGA-LGG dataset [80]). The second dataset (dataset2) consists of 3064 T1-weighted images with Glioma, Meningioma, and Pituitary brain tumor types, collected from 233 patients by Cheng et al. [81]. The second dataset can be retrieved from: https://figshare.com/articles/dataset/brain_tumor_dataset/1512427 (accessed on 28 June 2021).

Considering normal MRIs, an average of six middle MR slices were taken in the same intervals for every patient. The slices were utilized to differentiate healthy brains from brains with tumors. The identification of tumors was performed with the help of contrast infusion. Since the tumors can vary in size and their respective position in the brain, a different number of slices was used in various cases.

After the network has been trained, the classification of healthy and ill slices can be performed. The classifier network is able to recognize and distinguish abnormal images from healthy brains. This process has a two-fold benefit. First, it assists in identifying the presence of the tumor, and second, it pinpoints the approximate position of the tumorous tissue in the observed brain. Finally, it is possible to also determine the approximate size and the tumor grade.

The same image processing as in [9,10] is employed: pixel values of each image are normalized to scale [0,1] and, with the goal of increasing the size of the training dataset, the data augmentation technique is used.

**Figure 3:**
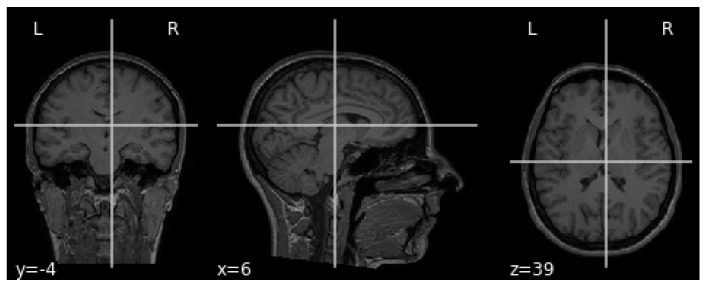
Axial MRI sample of a normal person with healthy brain.

The MRI requires the setup of specific parameters, including radio-frequency pulse and gradient. T1 and T2 sequences are usually used in practice, with both providing specific information about the observed tissue. This research uses the T1 sequence. To reduce the amount of images required for the healthy brain, six sections were chosen from MRI images. A healthy brain sample is shown in Figure 3.

**Figure 4:**
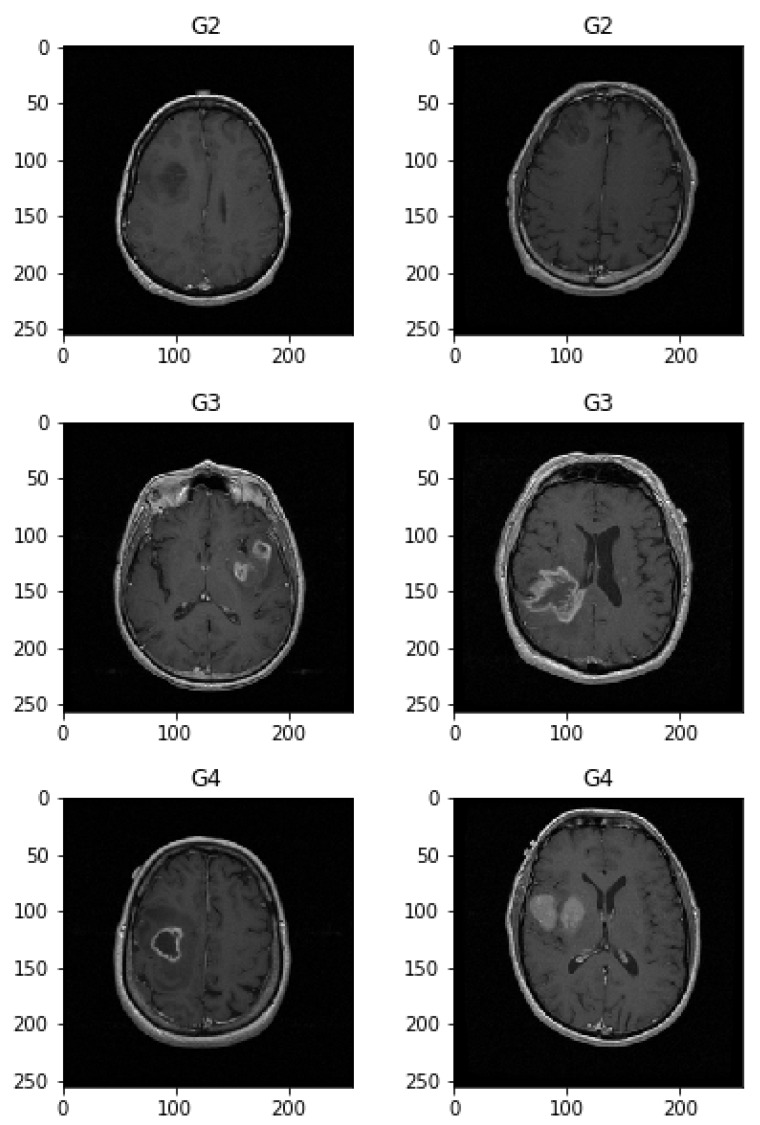
Axial MRI samples of different glioma grades: (**top**) glioma grade 2; (**middle**) glioma grade 3; (**bottom**) glioma grade 4.

**Figure 5:**
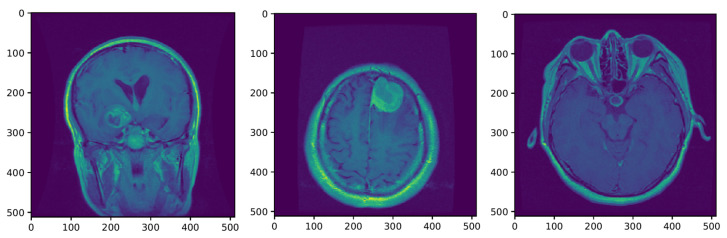
Axial brain samples from the public dataset given by Cheng in [81]: (**left**) glioma; (**center**) meningioma; (**right**) pituitary tumors.

To help distinguish the tumor tissue and more precisely determine the tumor borders, the patients are typically injected with a contrast solution (Gadolinium). These images can be utilized in the classification of the tumor grade. Figure 4 shows axial MRIs with Gadolinium infusion of three grades of glioma brain tumors. Finally, Figure 5 shows three brain tumor cases from the second dataset utilized in this research [81]. As mentioned, all employed images were normalized to the [0,1] interval, and their dimension was set to 128×128 pixels.

The network training on a bigger dataset is considered a successful approach for the generalization of and reduction in the over-fitting problem. Data augmentation refers to the process of creating fake data and the addition of these data to the dataset. In this study, some images were manipulated by utilizing random modifications of the original ones, including rotation by 10, 20, or 30 degrees in a random direction, translation by 15 pixels, re-scaling the image to 3/4 of the original dimensions, mirroring of the images, and combining those changes at the same time. The resulting manipulated images were included in the original datasets.

Consequently, the first dataset size is increased to 16,000 and consists of 8000 healthy and 8000 glioma brain tumor images with grades I-IV. Out of 16,000, 2000 images, which include 500 images from each category (normal, grade II, III and IV) were used for testing, while the remaining are used for training. The second dataset initially consisted of a total of 3064 axial images split into three categories: 708 meningioma tumors, 1426 glioma tumors, and 930 pituitary tumors. After performing the same data augmentation process, each of the three categories consists of 1521 images utilized for training, and 115 used for the test, the grand total of 4908 images. More details regarding the pre-processing phase and the split datasets can be captured from [9,10].

### 5.2. Basic Experimental Setup

The experimental setup (in terms of data pre-processing, split, validation criteria, and other simulation parameters) utilized in the proposed research is the same as in the referenced paper [9]. Moreover, as noted in Section 1, this research represents the continuation of the study shown in [10].

Hyper-parameters that have been put through the process of optimization included the number of convolutional layers and filters per layer, along with their respective size, employed activation function, pooling layer, number of fully connected layers and the hidden units in each, the number of dropout layers with dropout ratio, type of optimizer, and finally, the learning rate. Since the search space is enormous, it was limited by defining hyper-parameters’ possible values within the lower and upper bounds, which were suggested by the domain expert, as shown in [9]. A full list of hyper-parameters, together with their corresponding boundaries, is given in Table 7.

The number of convolutional and pooling layers (CL and PL) possible values are defined by the set {2,3,4,5,6}. The kernel size of the pooling layer has been fixed to 2×2. The filter size (FS) has been limited from 2 to 7. The number of filters (FN) was defined with the set of possible values {16,24,32,48,64,96,128}. The fully connected (FL) and dropout (DL) layers’ number was taken from the interval [1−3]. Concerning the number of hidden units (UN), this can take values from the set {128,192,256,384,512}.

The choice of the activation function (AF) was limited to four types, encoded with numeric values, as follows: ReLU = 1, ELU = 2, SELU = 3, and finally, LReLU = 4. The dropout rate (DR) and the learning rate (LR) can take any continuous value from the interval [0.1,0.5] and $\left[10^{-4},10^{-2}\right]$, respectively. This research, as in the ones proposed in [9,10], assumes the cross-entropy is utilized as a loss function with five possible optimizer (OF) options, encoded with integers (similar to the activation functions), and specified with the set {Adam=1, Adamax=2, Nadam=3, Adagard=4, Adadelta=5, SGD=6}. After defining all the hyper-parameters that are subjected to the CNN optimization process, the search space can be formulated as:

(30) S={CL,PL,FL,DL,FS,FN,UN,DR,AF,OF,LR}

where every element from the set refers to the corresponding hyper-parameter and its possible range of values. For more details, the interested reader can refer to the papers [9,10].

Therefore, according to (30), each solution from the population, which represents one possible CNN structure, is encoded as an array of size 11, and the whole population is represented as a matrix of N×11. Moreover, it should also be noted that some solution parameters are continuous, while some are discrete. Since the boundaries of integer parameters are relatively low, the search process is conducted by simply rounding the generated continuous to the nearest discrete value. Based on the previous studies, this is the most efficient way, with no additional computational burden [18,19].

The initial population is generated with random CNN architectures, followed by a calculation of the fitness for each CNN (candidate solution). The study proposed in [9,10] evolves 50 (N=50) possible network structures in 15 iterations T=15 and this setup, in the case of most metaheuristics approaches, yields a total number of 800 FFEs (N+N·T). However, as pointed out in Section 4, not all metaheuristics use the same number of FFEs in each iteration and, for that reason, in the proposed study, maxFFEs=800 is used as the termination condition instead of T=15. With this setup, similar conditions as in [9,10] were established. The eHHO control parameter ψ was set to 200, according to the expression shown in Section 4.1. However, since the CNN design experiment involves a much lower number of FFEs than CEC2019 simulations, the value for bt was determined empirically and set to 12, using the expression round((maxFFEs−ψ)/N).

The classification error rate on the test set represents the fitness function and the optimization goal is to find a CNN architecture with maximum accuracy (lowest error rate). The training set is split into the training and validation data using the 80−20 split rule. Each possible generated CNN structure (candidate solution) is trained on the training set and validated on the validation set in 100 epochs. With the goal of reducing computational costs, an early stopping condition is implemented, and if there is no improvement in validation accuracy in three subsequent epochs, the training will stop. When the training is finished, the fitness of the CNN structure (candidate individual) is calculated.

**Figure 6:**
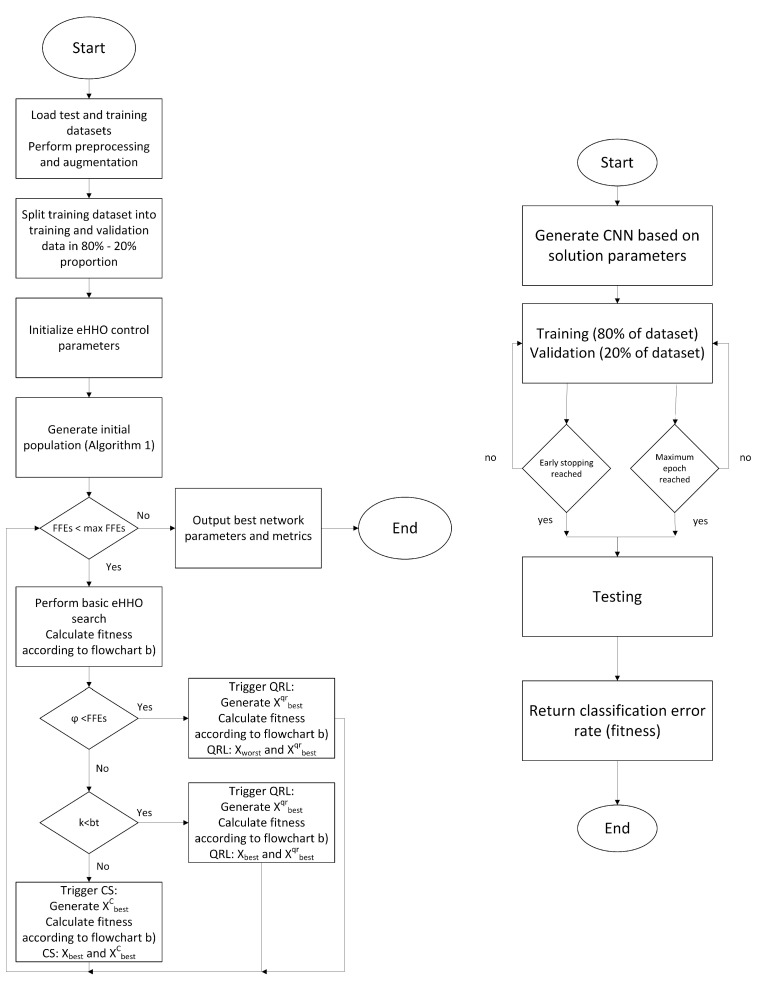
CNN + eHHO main flowchart (**left**); CNN + eHHO flowchart for calculating fitness (**right**).

Taking all into account, flowchart of proposed eHHO for CNN design (CNN + eHHO) is given in Figure 6.

### 5.3. Results, Comparative Analysis and Discussion

In this study, the performance of the proposed eHHO was compared with other state-of-the-art metaheuristics along with some classical machine learning approaches. All methods covered in the comparative analysis were implemented and tested for the purpose of this research under the same conditions to maintain an unbiased and fair comparison. Details of the experimental setup are explained in Section 5.2.

All metaheuristics were executed according to the flow diagram shown in Figure 6 in 10 independent runs, and the best results are reported. The adaptations of metaheuristics for CNN design are labeled with the prefix “CNN”, e.g., CNN + eHHO.

Metaheuristics were used to find a proper CNN structure within the boundaries of a search space, and this guided exploration is much more efficient than the exhaustive one. The search space is enormous and there are more than 1 million possible hyperparameter combinations. With this hyperparameter setup, a grid search cannot execute in a reasonable amount of computational time; however, by using metaheuristics, a satisfying CNN is generated by evaluating only 800 possible CNN architectures.

Due to the abovementioned argument, the grid search, as one of the most commonly used tools for searching near-optimal hyperparameter values for a specific classification/regression problem, is not included in the comparative analysis. Hoewver, the grid search method that consumes approximately the same processing time as the approach proposed in this study can only evaluate a few possible CNN structures and, as such, would be useless in practical implementation.

Besides the original HHO, the following metaheuristics were also included in analysis: genetic algorithm (GA) implementation proposed in [9], basic FA [82], modified firefly algorithm (mFA) [10], bat algorithm (BA) [15], EHO [67], WOA [17], SCA [70] and PSO [11]. All methods were tested with the control parameters’ values, suggested in the original papers, which are summarized in Table 8. For additional details, please refer to original manuscripts, where the abovementioned methods are proposed for the first time.

Moreover, to further facilitate comparative analysis and better evaluate the robustness of the proposed CNN + eHHO, some standard network structures (not evolved by metaheuristics) were also encompassed in comparative analysis: support vector machine (SVM) + recursive feature elimination (RFE) [83], Vanilla preprocessing + shallow CNN [84], CNN LeNet-5 [85], VGG19 [86] and DenseNet (Keras DenseNet201 instance) [87]. All these methods were instantiated with default parameters and trained for these particular datasets under the same conditions as metaheuristics.

Comparative analysis results in terms of classification accuracy between the proposed eHHO and original EHO, along with other state-of-the-art methods, are provided in Table 9. The results obtained for the GA and mFA differ from the ones reported in [9,10], respectively, because, in this study, the maxFFEs is taken as a termination condition.

Based on the results shown in Table 9, even the basic CNN + HHO obtained promising results compared to other methods. The CNN + HHO performed similarly to CNN + SCA and better than all other metaheuristics, except for the proposed CNN + eHHO and CNN + mFA. In both experiments (dataset1 and dataset2), CNN + EHO managed to generate a CNN that obtains 90.7% and 93.9 % accuracy for dataset1 and dataset2, respectively, which are the worst results among all the metaheuristics-based approaches. The other methods showed a similar performance for both datasets.

The proposed CNN + eHHO was established as a more robust and precise method compared to the CNN + HHO and other presented approaches for this specific task. For both datasets, the proposed CNN + eHHO managed to establish the greatest classification accuracy among all competing algorithms. The second best state-of-the-art approach is CNN + mFA [10]; however, CNN + eHHO outscores this for 2.6% in dataset1 and for 1.5% in dataset2 simulations in terms of accuracy. These performance improvements may not seem much; however, taking the sensitivity of medical diagnostics into consideration, even small enhancements in percent fractions can save lives by confirming glioma tumor cases in the early phases of progression.

Furthermore, some well-known CNN structures shown in the comparative analysis are deeper than the best-evolved solutions by metaheuristics, while the networks designed by metaheuristics achieved better classification accuracy. The metaheuristics enable an automated means of evolving possible CNN structures in an iterative guided manner by trying to minimize the classification error and, when a manual network crafting is performed, some of the possible hyper-parameters combinations can be examined.

**Figure 7:**
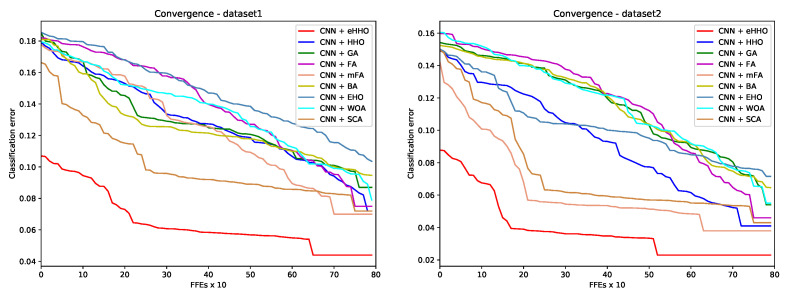
Classification error convergence speed graphs for dataset1 and dataset2 as a direct comparison between proposed eHHO and other metaheuristics.

Convergence speed graphs for one of the best runs for the classification error metrics of compared metaheuristics-based methods are provided in Figure 7. From the presented graphs, the superiority of CNN + eHHO in terms of solution quality (accuracy) and convergence speed can be observed. Moreover, it can be observed that the chaos-based initialization offers a significant advantage to CNN + eHHO upon generation of the initial population.

**Figure 8:**
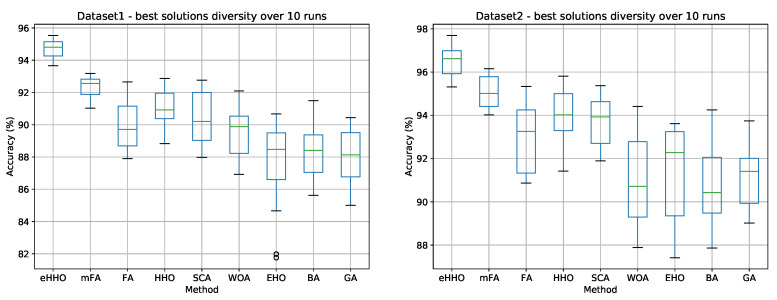
Diversity analysis for dataset1 and dataset2 as a direct comparison between proposed eHHO and other metaheuristics.

Besides the convergence speed, another very important indicator of an algorithm’s performance is diversity. By observing the best solutions’ diversity, generated at the end of the runs, the stability of the approach can be investigated. The diversity of the best solution (generated CNN structure) for all methods included in an analysis over 10 runs is shown in the box and whiskers diagrams presented in Figure 8.

From the presented graphs, it can be seen that the proposed CNN + eHHO and CNN + mFA exhibits stable behavior for both datasets, with a relatively low standard deviation value. Other methods, except for CNN + EHO, also perform well in terms of stability. However, the CNN + EHO approach exhibits higher standard deviation value, with an emphasized discrepancy between the best and worst solutions and, in the case of dataset1, even with some outliers.

**Figure 9:**
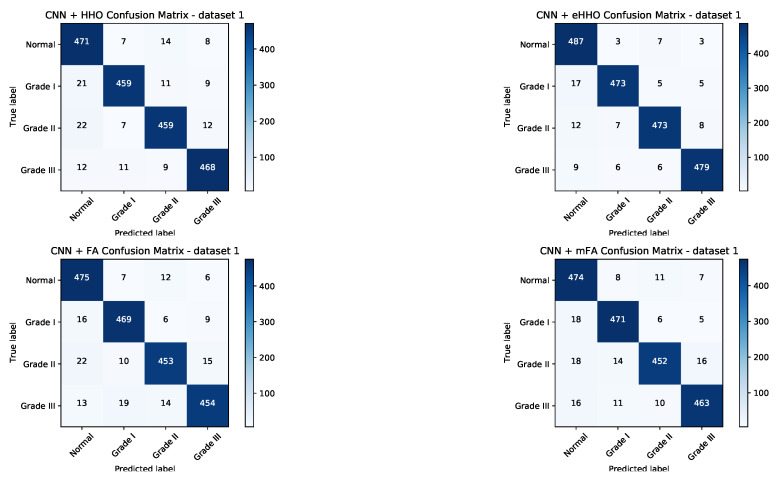
Confusion matrix for dataset1 with a direct comparison between proposed eHHO, HHO, FA and mFA approaches.

**Figure 10:**
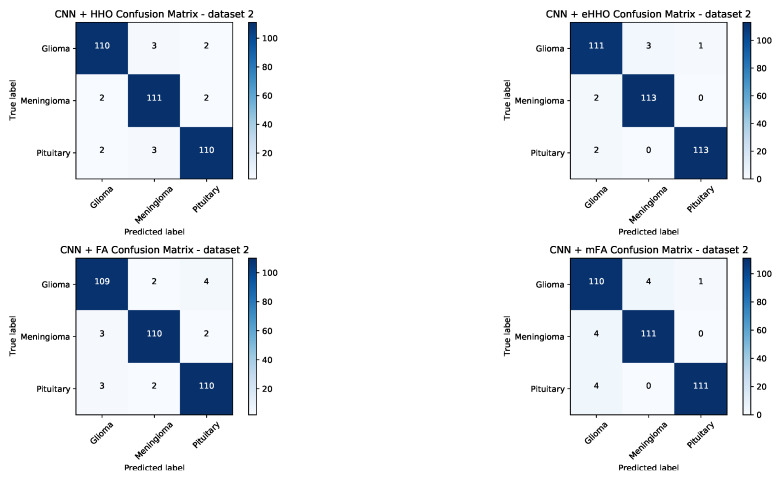
Confusion matrix for dataset2 with a direct comparison between proposed CNN + eHHO, CNN + HHO, CNN + FA and CNN + mFA approaches.

To provide better insight into the achieved results, the confusion matrices for both datasets have been generated for CNN + HHO, CNN + eHHO, CNN + FA and CNN + mFA approaches, and shown in Figure 9 and Figure 10.

In general, among all algorithms included in the comparative analysis, the two best approaches that were capable of generating the best-performing CNNs for both datasets are the proposed CNN + eHHO and CNN + mFA. Nonetheless, both methods for dataset1 and dataset1 managed to classify each tumor category with high accuracy. From the presented matrices, it can be unambiguously concluded that the CNN + eHHO performs better.

To further investigate the robustness of the two best-performing CNN classifiers, other performance metrics are presented for CNN + eHHO and CNN + mFA. These indicators are shown in Table 10 and Table 11. Both approaches were tested, with maxFFEs as the termination condition, and since FFEs is the most expensive operation, both algorithms take approximately the same computational time, and the complexity can be neglected. However, if the total number of iterations is taken as the termination condition, the mFA would be much more expensive than eHHO because, in each iteration, FA executes two loops and performs significantly more FFEs [54].

BExplanations for each abbreviation used in Table 10 and Table 11 are provided below:

- TP represents the true positives (correct predictions of positive class).
- TN represents the true negatives (correct predictions of negative class).
- FP denotes the false positives (incorrect predictions of positive class).
- FN denotes the false negatives (incorrect predictions of negative class).
- TPR stands for true positives rate, calculated as TP/(TP + FN). This value is also known as sensitivity, hit rate, or recall.
- TNR stands for true negatives rate, calculated as TN/(TN + FP). This value is also referred to as specificity.
- PPV denotes positive predictive value, calculated as TP/(TP + FP). This value is referred to as precision.
- NPV denotes negative predictive value, calculated as TN/(TN + FN).
- FPR stands for false positives rate, calculated as FP/(FP + TN).
- FNR stands for false negatives rate, calculated as FN/(TP + FN).
- FDR represents false discovery rate, calculated as FP/(TP + FP).
- ACC represents the overall accuracy, calculated as (TP + TN)/(P + N) = (TP + TN)/(TP + FP + FN + TN).

The results given in Table 10 and Table 11 show that the proposed CNN + eHHO approach is capable of achieving notably better results. When dataset1 is considered, the proposed CNN + eHHO achieves an accuracy of 0.974 for healthy brains (compared to the 0.961 achieved by CNN + mFA), 0.979 for grade II glioma (mFA achieved 0.969), 0.978 for grade III glioma (mFA scored 0.963), and 0.982 for grade IV glioma (compared to 0.967 achieved by CNN + mFA). When dataset2 is observed, the proposed eHHO achieved an accuracy of 0.977 for glioma tumors (compared to 0.962 achieved by mFA), 0.986 for meningioma tumors (CNN + mFA scored 0.977), and finally, 0.991 for pituitary tumors (while mFA achieved 0.986).

**Figure 11:**
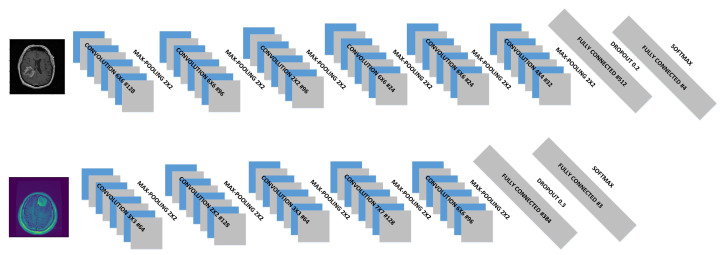
Examples of best performing network structures: dataset1 (top); dataset2 (bottom).

Some of the best-performing CNN structures generated by CNN + eHHO for both datasets are shown in Figure 11. Most of the best-performing CNN structures generated for dataset1 consist of six convolutional and max pooling layers with [64,128] filters, optimizers Adam or Adagard, LR∈[0.01,0.009] and ReLU activation function. However, the best-performing CNN architectures for dataset2 include five convolutional and max pooling layers with [24,128] filters, optimizers Adam or Adamx, LR∈[0.001,0.0009] and ReLU or LRelu activation functions.

**Figure 12:**
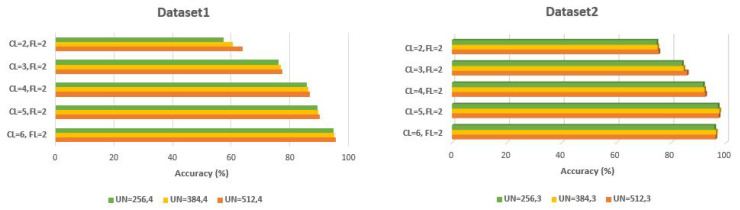
Impact of CL and UN in the dense layers on classification accuracy.

During simulations, various generated CNN structures evolved by CNN + eHHO were captured. It was determined that the highest impact on CNN performance for both datasets is the number of convolutional layers (CL) and the number of hidden units in the dense layers (UN), while most of the evolved network structures have two fully connected layers (FL=2). The impact of CL and UN on the classification performance of some generated networks for dataset1 and dataset2 is shown in Figure 12.

**Figure 13:**
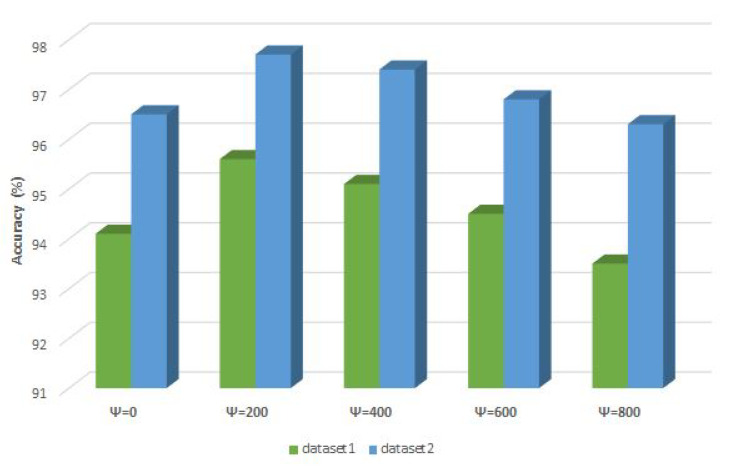
Effect of control parameter ψ on the CNN + eHHO performance.

Finally, as in the experiments with CEC2019 benchmarks (Section 4), to better determine the influence of control parameters ψ and bt on the CNN + eHHO performance, additional experiments with varying ψ values are conducted. The ψ was adjusted in increments of 200 FFEs (ψ=0,200,400,600 and 800), and the best solution’s accuracy (evolved CNN structure) is reported from a set of 10 independent runs. The bt value depends on ψ and was calculated according to the expression shown in Section 5.2. The results of this experiment for both datasets are summarized in Figure 13.

From the presented chart, it can be firmly concluded that with ψ set to 200 FFEs, the proposed CNN + eHHO obtains the best performance. The CNNs that obtain the lowest accuracy are generated with ψ=0 (magnified exploration) and ψ=800 (amplified exploitation). Moreover, it is interesting to note that in all additional experiments, the CNN + eHHO managed to find a CNN structure that outscores all other metaheuristics included in comparative analysis (Table 9).

## 6. Conclusions

The research shown in this manuscript proposes an enhanced version of HHO metaheuristics. The introduced eHHO algorithm was developed specifically to target the observed drawbacks of the original method by incorporating the chaotic mechanism and a novel, quasi-reflexive learning replacement strategy that enhances both the exploitation and exploration, with only a small additional overhead in terms of its computational complexity and new control parameters.

In the presented study, two types of experiments were performed. The proposed eHHO was first validated on a standard bound-constrained CEC2019 benchmark functions set, according to the firmly established practice in the recent computer science literature. The method was further validated on the practical problem of CNN design (hyperparameter optimization) and tested for classification of brain tumor MRI images.

The proposed method was compared to other state-of-the-art algorithms and obtained a superior performance in both conducted experiments. In simulations with CEC2019 instances, eHHO proved to be a robust approach and outscored all opponents in 8 out of 10 test functions. In the second experiment, CNN + eHHO managed to generate CNNs that are able to classify different grades of glioma tumors and two other common brain tumor variants with high precision by taking the raw MRI as input. Additionally, in the proposed study, other well-known swarm algorithms were also implemented for CNN design, and a broader comparative analysis was established. The CNN + eHHO substantially outscored all other approaches. In this way, the approach introduced in this study can be utilized as an additional option to help doctors in the early detection of gliomas and reduce the need for invasive procedures such as biopsy. It can also reduce the time for diagnostics, as the time needed to perform the classification is far shorter than the time required to analyze the biopsy.

Consequently, the contributions of this manuscript can be summarized as follows: basic HHO was improved and showed a superior performance compared to the other state-of-the-art methods for standard unconstrained problems and for the practical CNN design challenge. Furthermore, other swarm intelligence approaches that had not been tested before for this problem were implemented, and a performance comparison is provided of different metaheuristics for CNN hyperparameters’ optimization.

It should be stated here that the conducted research has some limitations. First, since the hyperparameters’ values were limited according to the domain expert from this area, as stated in [9] (otherwise, the search space would be infinite), not all possible structures were tested. Second, the eHHO algorithm that generates the possible CNN structures was not tested on generic datasets, such as CIFAR-10, USPS, Semeion, and MNIST, and this will be included in one of the future studies. Finally, since there are numerous new networks and approaches, not all of them could be included in the comparative analysis, as a long period of time would be needed for testing.

Possible directions of the future work in this area may include testing the proposed eHHO-driven CNN method for other MRI benchmark datasets and adapting the devised algorithm for tackling NP-hard challenges from other domains. As part of the future research, CNN structures evolved with the eHHO method could be evaluated by employing the semi-supervised learning approach to determine the impact of a smaller training sample set on the performance [88,89,90]. Finally, it is also possible to test other metaheuristics approaches in enhanced versions for the same glioma classification problem.

## Figures and Tables

**Table 1:** CEC 2019 benchmark characteristics.

No.	Functions	$F_{i}=F_{i}\left(x^{*}\right)$	D	Search Range
CEC01	Storn’s Chebyshev Polynomial Fitting Problem	1	9	[−8192, 8192]
CEC02	Inverse Hilbert Matrix Problem	1	16	[−16,384, 16,384]
CEC03	Lennard-Jones Minimum Energy Cluster	1	18	[−4, 4]
CEC04	Rastrigin’s Function	1	10	[−100, 100]
CEC05	Griewangk’s Function	1	10	[−100, 100]
CEC06	Weierstrass Function	1	10	[−100, 100]
CEC07	Modified Schwefel’s Function	1	10	[−100, 100]
CEC08	Expanded Schaffer’s F6 Function	1	10	[−100, 100]
CEC09	Happy Cat Function	1	10	[−100, 100]
CEC10	Ackley Function	1	10	[−100, 100]

**Table 2:** The eHHO control parameters overview.

Parameter Interpretation	Value
Population size (*N*)	50
Maximum number of FFEs (maxFFEs)	25,000
Dynamic behavior (ψ)	maxFFEs/4 = 6250
Best threshold (bt)	round((maxFFE−ψ)/(10·N)) = 38

**Table 3:** Evaluation of results achieved by different well-known metaheuristics on CEC2019 benchmark function set.

Function	Stats	EHOI	EHO	SCA	SSA	GOA	WOA	BBO	MFO	PSO	HHO	eHHO
CEC01	mean	$4.76\times 10^{4}$	$1.35\times 10^{7}$	$9.83\times 10^{9}$	$3.21\times 10^{9}$	$1.61\times 10^{10}$	$1.03\times 10^{10}$	$3.52\times 10^{10}$	$7.17\times 10^{9}$	$6.75\times 10^{11}$	$9.49\times 10^{4}$	$1.66\times 10^{3}$
	std	$2.14\times 10^{3}$	$7.91\times 10^{6}$	$6.95\times 10^{9}$	$1.42\times 10^{9}$	$8.99\times 10^{9}$	$9.14\times 10^{9}$	$2.32\times 10^{10}$	$8.69\times 10^{9}$	$2.34\times 10^{11}$	$2.53\times 10^{3}$	$1.23\times 10^{3}$
CEC02	mean	$1.70\times 10^{1}$	$1.72\times 10^{1}$	$1.75\times 10^{1}$	$1.73\times 10^{1}$	$1.74\times 10^{1}$	$1.73\times 10^{1}$	$8.87\times 10^{1}$	$1.74\times 10^{1}$	$8.56\times 10^{2}$	$2.42\times 10^{1}$	$1.25\times 10^{0}$
	std	$3.66\times 10^{-16}$	$7.29\times 10^{-15}$	$5.19\times 10^{-3}$	$6.55\times 10^{-5}$	$3.23\times 10^{-2}$	$1.95\times 10^{-3}$	$2.45\times 10^{1}$	$4.17\times 10^{-15}$	$3.87\times 10^{2}$	$1.33\times 10^{1}$	$7.44\times 10^{-18}$
CEC03	mean	$1.27\times 10^{1}$	$1.27\times 10^{1}$	$1.27\times 10^{1}$	$1.27\times 10^{1}$	$1.27\times 10^{1}$	$1.27\times 10^{1}$	$1.27\times 10^{1}$	$1.27\times 10^{1}$	$1.27\times 10^{1}$	$1.27\times 10^{1}$	$1.27\times 10^{1}$ *
	std	$3.95\times 10^{-16}$	$7.44\times 10^{-16}$	$3.25\times 10^{-4}$	$3.11\times 10^{-15}$	$6.47\times 10^{-4}$	$7.94\times 10^{-6}$	$5.25\times 10^{-7}$	$4.38\times 10^{-5}$	$4.12\times 10^{-4}$	$8.45\times 10^{-4}$	$5.17\times 10^{-12}$
CEC04	mean	$1.28\times 10^{1}$	$1.55\times 10^{1}$	$8.32\times 10^{2}$	$3.25\times 10^{1}$	$1.51\times 10^{2}$	$2.65\times 10^{2}$	$6.95\times 10^{1}$	$1.38\times 10^{2}$	$6.92\times 10^{1}$	$3.81\times 10^{1}$	$3.60\times 10^{0}$
	std	$4.26\times 10^{0}$	$8.52\times 10^{0}$	$3.85\times 10^{2}$	$1.09\times 10^{1}$	$1.13\times 10^{2}$	$1.39\times 10^{2}$	$2.99\times 10^{1}$	$1.15\times 10^{2}$	$5.43\times 10^{1}$	$2.55\times 10^{1}$	$0.62\times 10^{0}$
CEC05	mean	$1.05\times 10^{0}$	$1.07\times 10^{0}$	$2.23\times 10^{0}$	$1.35\times 10^{0}$	$1.33\times 10^{0}$	$1.67\times 10^{0}$	$1.31\times 10^{0}$	$1.13\times 10^{0}$	$1.55\times 10^{0}$	$1.14\times 10^{0}$	$1.01\times 10^{0}$
	std	$3.25\times 10^{-3}$	$2.41\times 10^{-2}$	$7.81\times 10^{-2}$	$2.33\times 10^{-1}$	$1.21\times 10^{-1}$	$3.86\times 10^{-2}$	$9.63\times 10^{-2}$	$6.56\times 10^{-2}$	$1.18\times 10^{-1}$	$4.73\times 10^{-2}$	$1.40\times 10^{-3}$
CEC06	mean	$8.33\times 10^{0}$	$9.45\times 10^{0}$	$1.04\times 10^{1}$	$3.79\times 10^{0}$	$6.19\times 10^{0}$	$9.14\times 10^{0}$	$5.78\times 10^{0}$	$4.92\times 10^{0}$	$1.03\times 10^{1}$	$9.96\times 10^{0}$	$1.75\times 10^{0}$
	std	$6.23\times 10^{-1}$	$1.31\times 10^{0}$	$8.15\times 10^{0}$	$1.23\times 10^{0}$	$1.33\times 10^{0}$	$1.05\times 10^{0}$	$2.99\times 10^{-1}$	$2.13\times 10^{0}$	$3.35\times 10^{0}$	$7.29\times 10^{-1}$	$6.59\times 10^{-3}$
CEC07	mean	$1.42\times 10^{2}$	$1.81\times 10^{2}$	$6.38\times 10^{2}$	$2.89\times 10^{2}$	$2.87\times 10^{2}$	$4.53\times 10^{2}$	$4.92\times 10^{0}$	$3.19\times 10^{2}$	$5.97\times 10^{2}$	$4.31\times 10^{2}$	$4.98\times 10^{0}$
	std	$1.13\times 10^{2}$	$1.51\times 10^{2}$	$2.78\times 10^{2}$	$2.25\times 10^{2}$	$1.75\times 10^{2}$	$2.25\times 10^{2}$	$1.21\times 10^{0}$	$2.15\times 10^{2}$	$1.89\times 10^{2}$	$1.95\times 10^{2}$	$3.29\times 10^{0}$
CEC08	mean	$2.69\times 10^{0}$	$3.15\times 10^{0}$	$5.77\times 10^{0}$	$5.08\times 10^{0}$	$5.49\times 10^{0}$	$5.75\times 10^{0}$	$4.81\times 10^{0}$	$5.45\times 10^{0}$	$5.10\times 10^{0}$	$3.73\times 10^{0}$	$1.44\times 10^{0}$
	std	$9.15\times 10^{-2}$	$1.44\times 10^{0}$	$7.29\times 10^{-1}$	$7.83\times 10^{-1}$	$5.14\times 10^{-1}$	$7.29\times 10^{-1}$	$1.03\times 10^{0}$	$5.62\times 10^{-1}$	$7.33\times 10^{-1}$	$5.99\times 10^{-1}$	$4.14\times 10^{-4}$
CEC09	mean	$2.29\times 10^{0}$	$2.41\times 10^{0}$	$8.75\times 10^{1}$	$2.38\times 10^{0}$	$2.45\times 10^{0}$	$5.16\times 10^{0}$	$3.75\times 10^{0}$	$2.46\times 10^{0}$	$2.65\times 10^{0}$	$4.78\times 10^{0}$	$1.63\times 10^{0}$
	std	$5.55\times 10^{-3}$	$2.18\times 10^{-2}$	$5.63\times 10^{1}$	$5.33\times 10^{-2}$	$6.41\times 10^{-2}$	$5.29\times 10^{-1}$	$3.14\times 10^{-1}$	$6.76\times 10^{-2}$	$8.45\times 10^{-2}$	$1.17\times 10^{-1}$	$6.77\times 10^{-5}$
CEC10	mean	$1.92\times 10^{1}$	$2.11\times 10^{1}$	$2.08\times 10^{1}$	$2.03\times 10^{1}$	$2.00\times 10^{1}$	$2.05\times 10^{1}$	$2.07\times 10^{1}$	$2.02\times 10^{1}$	$2.06\times 10^{1}$	$2.04\times 10^{1}$	$1.53\times 10^{1}$
	std	$3.49\times 10^{0}$	$7.29\times 10^{0}$	$6.45\times 10^{0}$	$8.19\times 10^{0}$	$6.67\times 10^{0}$	$3.52\times 10^{-1}$	$7.13\times 10^{0}$	$6.66\times 10^{-1}$	$9.81\times 10^{2}$	$1.33\times 10^{0}$	$1.14\times 10^{0}$

* All methods obtained the same result.

**Table 4:** Friedman test ranks for the compared algorithms over 10 CEC2019 functions.

Function	EHOI	EHO	SCA	SSA	GOA	WOA	BBO	MFO	PSO	HHO	eHHO
CEC01	2	4	7	5	9	8	10	6	11	3	1
CEC02	2	3	8	4.5	6.5	4.5	10	6.5	11	9	1
CEC03	6	6	6	6	6	6	6	6	6	6	6
CEC04	2	3	11	4	9	10	7	8	6	5	1
CEC05	2	3	11	8	7	10	6	4	9	5	1
CEC06	6	8	11	2	5	7	4	3	10	9	1
CEC07	3	4	11	6	5	9	1	7	10	8	2
CEC08	2	3	11	6	9	10	5	8	7	4	1
CEC09	2	4	11	3	5	10	8	6	7	9	1
CEC10	2	11	10	5	3	7	9	4	8	6	1
Average	2.9	4.9	9.7	4.95	6.45	8.15	6.6	5.85	8.5	6.4	1.6
Rank	2	3	11	4	7	9	8	5	10	6	1

**Table 5:** Results of the Holm’s step-down procedure.

Comparison	*p*-Value	Rank	0.05/(*k* − *i*)	0.1/(*k* − *i*)
eHHO vs. SCA	$2.37\times 10^{-8}$	0	0.005	0.01
eHHO vs. PSO	$1.64\times 10^{-6}$	1	0.005556	0.01111
eHHO vs. WOA	$5.03\times 10^{-6}$	2	0.006250	0.01250
eHHO vs. BBO	$3.74\times 10^{-4}$	3	0.007143	0.01429
eHHO vs. GOA	$5.38\times 10^{-4}$	4	0.008333	0.01667
eHHO vs. HHO	$6.06\times 10^{-4}$	5	0.010000	0.02000
eHHO vs. MFO	$2.08\times 10^{-3}$	6	0.012500	0.02500
eHHO vs. SSA	$1.19\times 10^{-2}$	7	0.016667	0.03333
eHHO vs. EHO	$1.30\times 10^{-2}$	8	0.025000	0.05000
eHHO vs. EHOI	$1.90\times 10^{-1}$	9	0.050000	0.10000

**Table 6:** Results with varying ψ value of proposed eHHO for CEC2019 benchmark suite.

Function	Statistics	ψ=0	ψ=6.250	ψ=12.500	ψ=18.750	ψ=25.000
CEC01	mean	$5.29\times 10^{5}$	$1.66\times 10^{3}$	$8.74\times 10^{3}$	$5.52\times 10^{4}$	$7.49\times 10^{5}$
CEC02	mean	$4.77\times 10^{1}$	$1.25\times 10^{0}$	$9.49\times 10^{0}$	$1.15\times 10^{1}$	$4.83\times 10^{1}$
CEC03	mean	$1.27\times 10^{1}$	$1.27\times 10^{1}$	$1.27\times 10^{1}$	$1.27\times 10^{1}$	$1.27\times 10^{1}$ *
CEC04	mean	$1.35\times 10^{1}$	$3.60\times 10^{0}$	$0.89\times 10^{1}$	$1.28\times 10^{1}$	$1.42\times 10^{1}$
CEC05	mean	$1.30\times 10^{0}$	$1.01\times 10^{0}$	$1.08\times 10^{0}$	$1.10\times 10^{0}$	$1.23\times 10^{0}$
CEC06	mean	$4.25\times 10^{0}$	$1.75\times 10^{0}$	$2.31\times 10^{0}$	$3.61\times 10^{0}$	$4.50\times 10^{0}$
CEC07	mean	$1.13\times 10^{1}$	$4.98\times 10^{0}$	$4.87\times 10^{0}$	$5.21\times 10^{0}$	$5.33\times 10^{0}$
CEC08	mean	$4.05\times 10^{0}$	$1.44\times 10^{0}$	$1.85\times 10^{0}$	$2.93\times 10^{0}$	$4.56\times 10^{0}$
CEC09	mean	$2.18\times 10^{0}$	$1.63\times 10^{0}$	$1.81\times 10^{0}$	$2.04\times 10^{0}$	$2.49\times 10^{0}$
CEC10	mean	$2.00\times 10^{1}$	$1.53\times 10^{1}$	$1.49\times 10^{1}$	$1.87\times 10^{1}$	$2.05\times 10^{1}$
Total best		0	7	2	0	0

* All methods obtained the same result.

**Table 7:** List of CNN hyper-parameters along with search space boundaries.

Hyperparameter Description	Boundaries
Number of convolutional layer (CL)	[2, 6]
Number of max-pooling layer (PL)	[2, 6]
Number of dense layer (FL)	[1, 3]
Number of dropout layers (DL)	[1, 3]
Filter size (FS)	[2, 7]
Number of filters (FN)	[16, 128]
Number of hidden units (UN)	[128, 512]
Dropout rate (DR)	[0.1, 0.5]
Activation function (AF)	[1, 4]
Optimizer (OF)	[1, 6]
Learning rate (LR)	[0.01, 0.0001]

**Table 8:** Control parameters’ setup for metaheuristics included in analysis.

Algorithm	Parameters
GA [9]	$p_{c}=0.5$, $p_{m}=0.2$
FA [82]	α=0.5, β=0.2, γ=1.0
mFA [10]	α=0.5, β=0.2, γ=1.0, TL=20FFEs
BA [15]	$Q_{min}=0$, $Q_{max}=2$, A=0.5, r=0.5
EHO [67]	$no_{c}lan=5$, α=0.5, β=0.1, $no_{e}lite=2$
WOA [17]	$a_{1}$ linearly decreasing from 2 to 0, $a_{2}$ linearly decreasing from −1 to −2, *b* = 1
SCA [70]	a=2, $r_{1}$ linearly decreasing from 2 to 0

**Table 9:** MRI tumor grades classification comparative analysis.

Approach				Accuracy	
Case Study I (dataset1) – Glioma Grade II/Grade III/Grade IV					
SVM + RFE [83]				62.5%	
Vanilla pre-processing + shallow CNN [84]				82.8%	
CNN LeNet-5 [85]				63.6%	
VGG19 [86]				87.3%	
DenseNet [87]				88.1%	
CNN + GA [9]				91.3%	
CNN + FA [82]				92.5%	
CNN + mFA [10]				93.0%	
CNN + BA [15]				91.6%	
CNN + EHO [67]				90.7%	
CNN + WOA [17]				92.1%	
CNN + SCA [70]				92.8%	
CNN + HHO				92.8%	
CNN + eHHO				**95.6%**	
Case Study II (dataset2) – Glioma/Meningioma/Pituitary					
SVM + RFE [83]				71.2%	
Vanilla preprocessing + shallow CNN [84]				91.4%	
CNN LeNet-5 [85]				74.9%	
VGG19 [86]				92.6%	
DenseNet [87]				92.7%	
CNN + GA [9]				94.6%	
CNN + FA [10]				95.4%	
CNN + mFA [10]				96.2%	
CNN + BA [15]				94.7%	
CNN + EHO [67]				93.9%	
CNN + WOA [17]				94.5%	
CNN + SCA [70]				95.7%	
CNN + HHO				95.9%	
CNN + eHHO				**97.7%**	

**Table 10:** MRI brain tumor classification performance metrics for proposed CNN + eHHO approach.

		TP	FP	TN	FN	TPR	TNR	PPV	NPV	FPR	FNR	FDR	ACC	F1
DATASET 1														
Normal		487	38	1462	13	0.974	0.974	0.928	0.991	0.025	0.026	0.072	0.974	0.950
Grade II		473	16	1484	27	0.946	0.989	0.967	0.982	0.011	0.054	0.033	0.979	0.957
Grade III		473	18	1482	27	0.946	0.988	0.963	0.982	0.012	0.054	0.037	0.978	0.955
Grade IV		479	16	1484	21	0.958	0.989	0.967	0.986	0.011	0.042	0.032	0.982	0.963
**DATASET 2**														
Glioma		111	4	226	4	0.965	0.983	0.965	0.983	0.017	0.035	0.035	0.977	0.965
Meningioma		113	3	227	2	0.983	0.987	0.974	0.991	0.013	0.017	0.026	0.986	0.978
Pituitary		113	1	229	2	0.983	0.996	0.991	0.991	0.004	0.017	0.009	0.991	0.987

**Table 11:** MRI brain tumor classification performance metrics for CNN + mFA approach [10].

		TP	FP	TN	FN	TPR	TNR	PPV	NPV	FPR	FNR	FDR	ACC	F1
DATASET 1														
Normal		474	52	1448	26	0.948	0.965	0.901	0.982	0.035	0.052	0.099	0.961	0.924
Grade II		471	33	1467	29	0.942	0.978	0.935	0.981	0.022	0.058	0.065	0.969	0.938
Grade III		452	27	1473	48	0.904	0.982	0.944	0.968	0.018	0.096	0.056	0.963	0.924
Grade IV		463	28	1472	37	0.926	0.981	0.943	0.975	0.019	0.074	0.057	0.967	0.934
**DATASET 2**														
Glioma		110	8	222	5	0.957	0.965	0.932	0.978	0.035	0.043	0.068	0.962	0.944
Meningioma		111	4	226	4	0.965	0.983	0.965	0.983	0.017	0.035	0.035	0.977	0.965
Pituitary		111	1	229	4	0.965	0.996	0.991	0.983	0.004	0.035	0.009	0.986	0.978
